# Safety, effectiveness and treatment patterns of sodium zirconium cyclosilicate for hyperkalemia management in China: actualize study

**DOI:** 10.3389/fphar.2026.1744687

**Published:** 2026-05-14

**Authors:** Nan Shen, Xin Zeng, Hua Xie, Lihong Zhang, Jing Yang, Yongqiang Lin, Xinyu Liu, Xudong Cai, Juan Cao, Qiang Zhu, Xun Luo, Xin Wan, Henglan Wu, Jianming Ye, Chunyan Shan, Yifan Wu, Yanping Cao, Yang Lin, Xiaoyong Yu, Huimin Wang, Jingdong He, Shaojiang Tian, Fenglei Wu, Xinxin Jiang, Lu Li, Li Zuo, Zhaohua Wang, Changying Xing, Xun Yin, Jianrong Zhao, Cong Ma, Gang Long, Qing Li, Yao Hu, Yifan Shi, Hongli Lin

**Affiliations:** 1 The First Affiliated Hospital of Dalian Medical University, Dalian, Liaoning, China; 2 Graduate School of Dalian Medical University, Dalian, China; 3 Dalian Ruikaer Renal Disease Hospital, Dalian, China; 4 The First Hospital of Hebei Medical University, Shijiazhuang, Hebei, China; 5 Hefei First People’s Hospital, Hefei, Anhui, China; 6 Wenzhou Integrated Chinese and Western Medicine Hospital, Wenzhou, Zhejiang, China; 7 Nanyang Central Hospital, Nanyang, Henan, China; 8 Ningbo Traditional Chinese Medicine Hospital, Ningbo, Zhejiang, China; 9 Taixing People’s Hospital, Taizhou, Jiangsu, China; 10 Xinghua People’s hospital, Xinghua, China; 11 Hunan Provincial People’s Hospital, Changsha, Hunan, China; 12 The First Hospital of Nanjing, Nanjing, Jiangsu, China; 13 The First Hospital of Jiaxing, Jiaxing, Zhejiang, China; 14 The First People’s Hospital of Kunshan, Suzhou, Jiangsu, China; 15 Chu Hsien-I Memorial Hospital of Tianjin Medical University, Tianjin, China; 16 Guangdong Provincial Hospital of Traditional Chinese Medicine, Guangzhou, Guangdong, China; 17 Handan First Hospital, Handan, China; 18 Linfen Central Hospital, Linfen, Shanxi, China; 19 Shanxi Provincial Hospital of Chinese Medicine, Taiyuan, Shanxi, China; 20 Liaoning Health Industry Group Bensteel General Hospital, Benxi, Liaoning, China; 21 Nuclear Industry 416 Hospital, Chengdu, Sichuan, China; 22 Shiyan People’s Hospital, Shiyan, Hubei, China; 23 Qidong People’s Hospital Affiliated Qidong Hospital of Nantong University, Jiangsu, China; 24 Sandun District of Zhejiang Hospital, Hangzhou, Zhejiang, China; 25 The First Affiliated Hospital of Xi’an Medical University, Xi’an, Shaanxi, China; 26 Peking University People’s Hospital, Beijing, China; 27 Taian City Central Hospital, Tai’an, Shandong, China; 28 Jiangsu Province Official Hospital, Nanjing, Jiangsu, China; 29 Changshu No.2 People’s Hospital, Suzhou, Jiangsu, China; 30 The Affiliated Hospital of Inner Mongolia Medical University, Hohhot, Inner Mongolia, China; 31 Anshan Central Hospital, Anshan, Liaoning, China; 32 Tianjin People’s Hospital, Tianjin, China; 33 Tianjin Teda Hospital, Tianjin, China; 34 Clinical Medical College & Affiliated Hospital of Chengdu University, Chengdu, Sichuan, China; 35 AstraZeneca Investment China Co, Medical Affairs, Shanghai, China

**Keywords:** China, hyperkalemia, real-world data, safety and effectiveness, sodium zirconium cyclosilicate

## Abstract

**Introduction:**

This real-world analysis aimed to evaluate the primary endpoint safety profile and the secondary effectiveness and treatment patterns of sodium zirconium cyclosilicate (SZC) for hyperkalemia management in China, addressing the current evidence gap on SZC use in clinical practice.

**Methods:**

This multi-center, prospective, non-interventional cohort study enrolled patients (aged ≥18 years), including new and ongoing SZC users, from 34-sites. Treatment was categorized into correction-phase (FAS-P1) and maintenance phase (FAS-P2, new and ongoing users). The subgroup analysis was performed in patients undergoing hemodialysis (full analysis set-hemodialysis [FAS-H]). The primary objective included the safety of SZC whereas treatment patterns and effectiveness were the secondary objectives.

**Results:**

Of the 1,000 enrolled patients, 442, 878, and 474 were included in FAS-P1, FAS-P2, and FAS-H subgroups, respectively. The most frequently used SZC dosage was 5 g once-daily in FAS-P (42.1%), and FAS-P2 (37.7%). In the FAS-P1 and FAS-P2 groups, 64% and 65.4% of patients reported AEs; 26.9% and 30.8% reported SAEs. The serum potassium (sK^+^) was reduced from 5.8 to 5.0 mmol/L in FAS-P1. The mean “with-in” sK^+^ levels over 6-month was 5.1 mmol/L in FAS-P2, of which the new-user group were slightly higher vs. ongoing group of FAS-P2 with a mean sK^+^ of 5.2 and 5.0 mmol/L, respectively. Furthermore, SZC showed good efficacy in patients with different chronic kidney disease stage subgroups of FAS-P2.

**Conclusion:**

In real-world clinical practice, no new safety issues of SZC were identified. SZC was effective in lowering sK^+^ levels in the correction-phase and remained stable during the maintenance-phase.

## 1 Introduction

Hyperkalemia (HK), defined as high levels of serum potassium ([sK^+^] >5.0 mmol/L), can increase the risk of all-cause mortality, particularly in patients with chronic kidney disease (CKD), heart failure (HF), or diabetes (Collins et al., 2017; Clase et al., 2020). The condition can be classified as mild (5.1–5.9 mmol/L), moderate (6.0–6.4 mmol/L), or severe (≥6.5 mmol/L); (Sevamontree et al., 2024); however, symptoms can vary based on the rate of change, and the condition can be severe even at lower levels with sudden shifts (Abou Sherif et al., 2024). The urgency of treatment depends on the symptoms, serum levels, and causes of HK (Hunter and Bailey, 2019).

HK is prevalent in patients with CKD and HF (22.9% of patients with CKD and 12.5% of patients with HF) (Bian, 2021). Treatment with renin-angiotensin-aldosterone system inhibitors (RAASi) in these patients can further worsen HK (Polson et al., 2017; Svensson et al., 2024). Concomitant therapies such as RAASi, particularly mineralocorticoid receptor antagonists like spironolactone, significantly influence serum potassium levels (Georgianos and Agarwal, 2021; Chen et al., 2025). Although these therapies are essential for slowing CKD and HF progression, they increase the risk of hyperkalemia and often require dose reduction or discontinuation in routine practice. Standard acute management of hyperkalemia typically includes intravenous calcium, insulin with glucose, β-agonists, and diuretics, in accordance with international and national guideline recommendations (Elliott et al., 2010; Palmer et al., 2021; Alzahrani et al., 2024). Conventional potassium binders such as calcium polystyrene sulfonate are used cautiously because of concerns regarding gastrointestinal safety. These are prescribed only for short-term use because of the controversy regarding the long-term effects of calcium polystyrene sulfonate leading to colonic necrosis (Tamargo et al., 2018). There is no established long-term management of sK^+^ levels besides lifestyle interventions in the current clinical practice (Polson et al., 2017). Therefore, there is a need for additional agents that can safely treat HK in patients with acute and chronic HK.

In this regard, sodium zirconium cyclosilicate (SZC) was approved in China for the treatment of HK in December 2019. It is an oral, non-absorbable potassium binder that selectively binds to potassium ions and exchanges them for hydrogen and sodium ions throughout the entire gastrointestinal (GI) tract (Fujioka et al., 2023). This process minimizes the concentration of free potassium in the GI lumen, thereby reducing sK^+^ levels and increasing the excretion of fecal potassium to resolve HK (high potassium levels). Several clinical trials have shown that SZC can rapidly lower sK^+^ levels in patients with HK, making it a promising alternative to insulin and glucose (Shrestha et al., 2021; Fujioka et al., 2023). Beyond China, SZC has demonstrated rapid potassium-lowering efficacy and a consistent safety profile in multiple international phase 2 and 3 trials involving patients with CKD and heart failure (Ni et al., 2023; Shen et al., 2024). Long-term extension studies and real-world analyses from the United States, Europe, and Japan further support its tolerability and effectiveness across broader clinical populations.

As compared with the controlled phase 2/3 studies, post-marketing observational studies are essential to provide data regarding the efficacy and safety profile of a drug closer to the clinical practice in the real-world. However, studies that have evaluated the real-world treatment profile of SZC’s in the Chinese population are lacking. Here, we report the final results of safety, effectiveness, and treatment patterns of SZC in patients with HK. This real-world study was designed with a primary objective to evaluate the safety of SZC, and secondary objectives to assess its effectiveness in controlling serum potassium levels and to describe treatment patterns in routine clinical practice.

## 2 Methods

### 2.1 Study design

This was a multi-center, prospective, observational cohort study conducted across 34 centers in China. The detailed methodology has been published previously (Shen et al., 2023; 2024).

### 2.2 Study participants

The study included patients aged ≥18 years with at least one sK^+^ levels >5.0 mmol/L within 1 year before enrollment, currently receiving SZC treatment with a physician’s prescription, with or without hemodialysis. Patients were categorized into new SZC users (patients without SZC treatment within 7 days before enrollment, receiving SZC on the study enrollment day) and ongoing users (patients with SZC treatment within 7 days before the study enrollment day and continued the treatment even after enrollment). The distinction between new and ongoing SZC users was applied to differentiate the correction-phase from the maintenance phase of potassium management. New users contribute to the early treatment window where acute potassium reduction is expected, whereas ongoing users represent stable, longer-term management. This stratification enables evaluation of SZC performance across both the initial correction and maintenance periods over the 6-month follow-up.

All patients were followed up at the 1st, 3rd and the 6th month from the study enrollment day, with an additional follow-up visit on the third day for patients in the new SZC user group. Data including the safety and effectiveness information, sK^+^ levels, SZC treatment details, and any other relevant data were recorded at each visit from day 1 to month 6, if available. Additional sK^+^ tests were conducted as needed for potassium monitoring.

The study adhered to ethical guidelines and received approval from the Ethical Committee of the First Affiliated Hospital of Dalian Medical University (approval number: YJ-JG-YW-2020). An informed consent was obtained from all participants before the study initiation.

### 2.3 Full analysis sets definitions

Two full analysis sets were defined for the study. FAS-P1 included all new SZC users who received at least one dose of SZC during the first treatment period (Days 1–3 after initiation), representing the correction-phase. FAS-P2 included all ongoing SZC users who were receiving SZC at enrollment and new users who received at least one dose after the completion of the initial 3-day correction-phase. This population represents the maintenance phase. These groups were used for all primary and secondary endpoint analyses.

### 2.4 Treatment regimen

The initial recommended dose of SZC (10 g) was administered as an oral suspension in water 3 times a day (TID) for the first 2 days after enrollment. However, as this was a prospective, non-interventional, real-world study, the dosing regimen was not mandated by the study protocol. The actual SZC dose, frequency, and duration were determined at the discretion of the treating physician based on routine clinical practice. Patients were switched to maintenance therapy after they reached normokalemia with a SZC dosage that ranged from 5 g on alternate days to 10 g each day (doses were adjusted between 5 and 15 g once daily [QD] for patients undergoing dialysis) according to the Chinese prescription label. The dosage and duration of SZC treatment were decided by the treating physician. Discontinuation of the SZC treatment included patients who were not receiving SZC treatment for ≥7 days.

### 2.5 Study objectives and endpoints

The primary objective was to evaluate the safety of SZC by assessing AEs, SAEs and discontinuations due to AEs. Adverse events (AEs) were defined as any untoward medical occurrence observed during SZC therapy, irrespective of whether a causal relationship with the drug was suspected. Serious adverse events (SAEs) included events resulting in hospitalization, disability, death, or considered medically significant. Safety data were collected prospectively during each follow-up visit using patient interviews, medical record review, and laboratory results. Causality between SZC and each AE was assessed by the treating investigator based on clinical judgment, temporal relationship, alternative etiologies, and response to dose modification. Events judged as possibly, probably, or definitely related were classified as causally related AEs. For safety, all AEs, SAEs, causally related events, and discontinuations due to AEs were systematically evaluated for both FAS-P1 and FAS-P2. Secondary objectives included assessing effectiveness through changes in serum potassium in the full population and characterizing SZC treatment patterns, including dose exposure and modifications.

### 2.6 Data source and collection

The patient data were prospectively collected from 34 hospitals in China. It also included data from electronic or paper medical records, local laboratory testing records, and safety data according to standard clinical practice. Data on patient demographics, medical history, chronic conditions, and concomitant treatment were collected as well. Parameters such as vital signs, physical examinations, and various laboratory examinations including biochemical parameters were assessed at each visit until 6 months after the SZC treatment.

### 2.7 Statistical analyses

Analyses were split by a period, and 2 full analysis sets (FAS) were defined for each period: full analysis set-first period ([FAS-P1], consisting of all new users who received at least 1 dose of SZC during the first period of 1‒3 days post SZC initiation) and full analysis set-second period ([FAS-P2], consisting of all patients in the ongoing user group who received at least 1 dose of SZC post enrollment and new users who received at least 1 dose of SZC post completion of the initial period). The primary endpoints (AEs, SAEs, DAEs, and specific AEs) were summarized in terms of the number and percentage of patients, as well as incidence rates, and cumulative incidences, along with the 95% confidence interval (CI), based on FAS-P1 and FAS-P2. The average SZC daily dosage, frequency of different SZC dosages, duration of SZC treatment, dose changes, and reasons for any dose changes, were summarized descriptively for FAS-P1 and FAS-P2. Change in sK^+^ levels between visit 1 (V1) and visit 2 (V2) and the corresponding 95% CI were calculated based on FAS-P1. Average (within patient) sK^+^ levels during the second period and the proportion of patients with normokalemia were summarized along with 95% CI based on FAS-P2. In addition, supplementary analyses were carried out for patients undergoing hemodialysis (FAS-H) at the time of study enrollment. Protocol deviations were defined as any departure from study procedures, eligibility requirements, visit schedules, or data-collection processes specified in the protocol. Major deviations included incorrect population assignment, missing key safety or efficacy data, or violations affecting endpoint interpretation. All deviations were recorded and reviewed before the database lock. All analyses were performed using the SAS version 9.4.

## 3 Results

### 3.1 Study follow-up and attrition

**FIGURE 1:**
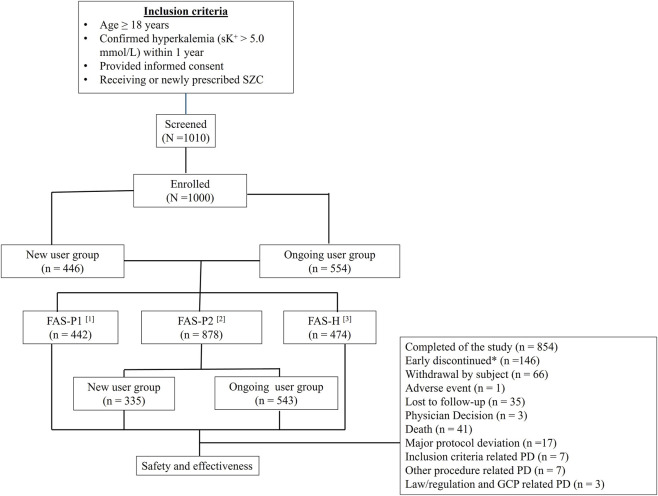
Patient disposition. Percentages were based on the total number of enrolled patients. ^[1]^FAS-P1: Consists of all new users who received at least 1 dose of SZC during the first period of 1–3 days post SZC initiation. ^[2]^FAS-P2: Consist of all patients in the ongoing user group who took at least 1 dose of SZC post enrollment, and new users received at least one dose of SZC post the completion of the initial period. ^[3]^FAS-H: Consist of patients who were undergoing hemodialysis at study enrollment. *Discontinuation of SZC was physician-determined in routine practice. Abbreviations: FAS-H, full analysis set-hemodialysis; FAS-P1, full analysis set-first period; FAS-P2, full analysis set-second period; GCP, Good clinical practice; PD, protocol deviation.

Overall, 1,000 patients from 34 sites were enrolled and categorized into new users (n = 446) and ongoing users (n = 554). Among them, 146 patients (14.6%) discontinued the study because of various reasons as outlined in Figure 1. Of all the enrolled patients, 442 were included in the FAS-P1, 878 in the FAS-P2, and 474 in the FAS-H. Overall, 17 patients (1.7%) had at least 1 major protocol deviation (PD). This study did not evaluate SZC for the emergency management of acute, life-threatening hyperkalemia. Patients requiring immediate stabilizing interventions (e.g., intravenous calcium, insulin–glucose therapy, or emergent dialysis) were treated according to routine clinical practice, and SZC initiation in such contexts was not part of the study design. Therefore, the analysis reflects non-acute, real-world use of SZC for potassium control.

### 3.2 Demographic and baseline characteristics

The mean (SD) age of all enrolled patients was 57.6 (14.2) years and 61.4% of them were male. The mean baseline body mass index (BMI) was 23.7 (3.7) kg/m2 and 388 patients had CKD at the enrollment, with the majority in stage 5 (n = 272, 70.1%). Of the 1,000 enrolled patients, 474 (47.4%) were undergoing hemodialysis at the time of enrollment (Table 1). Comorbidities relevant to hyperkalemia risk were frequently observed in this population, reflecting the complex clinical background of patients requiring SZC therapy. Comorbidities and concomitant medication use reflected the complex cardiovascular and renal disease burden of the study population. Cardiovascular conditions, including hypertension, coronary artery disease, and heart failure, were commonly documented across study sites. Consistent with routine management of chronic kidney disease and cardiorenal syndromes, a substantial proportion of patients received concomitant cardiovascular therapies that are known to influence serum potassium homeostasis. These included renin–angiotensin–aldosterone system inhibitors (angiotensin-converting enzyme inhibitors and angiotensin receptor blockers) and mineralocorticoid receptor antagonists, which are cornerstone treatments for heart failure but are frequently limited by hyperkalemia risk. Additional commonly prescribed therapies comprised calcium channel blockers, antianemic agents, and medications for metabolic and gastrointestinal disorders. Nearly half of the patients were undergoing hemodialysis at enrollment, with additional renal replacement modalities, including peritoneal dialysis, also reported. A detailed breakdown of baseline comorbidities and concomitant therapies is provided in Supplementary Table S1, further highlighting the clinically heterogeneous, high-risk nature of this real-world cohort.

**TABLE 1:** Demographic and baseline characteristics.

Characteristics category/statistic	Combining new user group and ongoing user group Total (N = 1,000)*	FAS-P1 Total (N = 442)	FAS-P2 Total (N = 878)	Hemodialysis at enrollment subgroup analysis (FAS-H) Total (N = 474)
Age (years)				
Mean (SD)	57.6 (14.2)	57.4 (13.8)	57.3 (14.2)	54.6 (13.0)
Age group (years, n [%])				
<65	658 (65.8)	297 (67.2)	584 (66.5)	361 (76.2)
≥65	342 (34.2)	145 (32.8)	294 (33.5)	113 (23.8)
Sex, n (%)				
Male	614 (61.4)	268 (60.6)	531 (60.5)	278 (58.6)
Female	386 (38.6)	174 (39.4)	347 (39.5)	196 (41.4)
Ethnicity, n (%)				
Hispanic or latino	0	0	0	0
Not hispanic or latino	1,000 (100)	442 (100)	878 (100)	474 (100)
BMI (kg/m^2^)^[1]^				
n (missing)	929 (71)	413 (29)	815 (63)	452 (22)
Mean (SD)	23.65 (3.7)	23.88 (3.7)	23.57 (3.7)	22.92 (3.7)
Hyperkalemia diagnosis code recorded at enrollment^#^, n (%)				
Yes	1,000 (100)	167 (37.8)	878 (100)	474 (100)
No	0	275 (62.2)	0	0
On hemodialysis, n (%)				
n (missing)	1,000 (0)	442 (0)	878 (0)	-
Yes	474 (47.4)	167 (37.8)	428 (48.7)	-
No	526 (52.6)	275 (62.2)	450 (51.3)	-
CKD staging at enrollment, n (%)				
n (missing)	388 (612)	201 (241)	317 (561)	125 (349)
Stage 1	0	0	0	0
Stage 2	6 (1.5)	3 (1.5)	6 (1.9)	0
Stage 3	37 (9.5)	19 (9.5)	31 (9.8)	1 (0.8)
Stage 4	73 (18.8)	44 (21.9)	63 (19.9)	0
Stage 5	272 (70.1)	135 (67.2)	217 (68.5)	124 (99.2)

Percentages were based on the number of patients in the new user groups, and ongoing groups of, FAS-P1, FAS-P2 and FAS-H, who provided corresponding value to the variable.

* Treatment duration over the second period for the new users (days) = Date of last SZC, dose Date of second visit +1 day of interruption.

* Treatment duration over the second period for the ongoing users (days) = Date of last SZC, dose Date of first visit +1 day of interruption.

#The variable ‘diagnosed with hyperkalemia’ reflects only whether a hyperkalemia diagnosis code was entered at the enrollment visit. All participants fulfilled the biochemical inclusion criterion of sK^+^ >5.0 mmol/L within the prior 12 months. Values marked ‘No’ therefore represent missing diagnosis codes rather than absence of hyperkalemia.

^[1]^BMI, weight (kg)/height (m)^2^,

rounded to 1 decimal place.

Percentages are calculated based on the number of patients with available data for each variable (denominator provided in the table). CKD, staging, comorbidity information, and some concomitant therapy records were available only for participants with non-missing documentation at enrollment. Hemodialysis status, however, was recorded for all enrolled patients. No imputation was performed for missing data; Abbreviations BMI, body mass index; FAS-H, full analysis set-hemodialysis; FAS-P1, full analysis set-first period; FAS-P2, full analysis set-second period; SD, standard deviation.

The baseline demographic characteristics of patients in the FAS-P1 and FAS-P2 are presented in Table 1. Among those with an available value of CKD stage at enrollment in FAS-P1 and FAS-P2, the majority of the patients were reported as having CKD stage 5 (n = 135, [67.2%] and n = 217, [68.5%], respectively). At study enrollment, 167 patients (37.8%) and 428 patients (48.7%) were undergoing hemodialysis in the FAS-P1 and FAS-P2, respectively.

At the enrollment, majority of the new user and ongoing user groups in FAS-P2, that is 134 (n = 82, 61.2%) and 183 patients (n = 135, 73.8%), respectively, had CKD stage 5. At the time of enrollment, 129 patients (38.5%) and 299 patients (55.1%) of the new user group and ongoing user group, respectively, were undergoing hemodialysis. The demographics and baseline characteristics of 474 patients in FAS-H were similar to the overall results (Table 1). The number of patients undergoing hemodialysis at enrollment (n = 474) appears larger than the number of patients with documented CKD staging because CKD stage was available only for individuals with non-missing staging data in the medical record, whereas hemodialysis status was captured for all enrolled patients. As a result, the CKD staging denominators shown in Table 1 reflect only those with available staging values, while dialysis status represents complete data for the full cohort. Among patients with recorded CKD stage, the majority were classified as stage 5, which is consistent with the high proportion of participants receiving hemodialysis.

Medications such as calcium channel blockers, antianemic drugs, alimentary tract and metabolism products along with RAASi were the commonly used by the patients in both groups. They underwent concomitant procedures such as hemodialysis (48.2% and 59.0%), peritoneal dialysis (9.5% and 7.5%), and symptomatic treatments (2.7% and 3.4%), respectively.

The variable “diagnosed with hyperkalemia” in Table 1 reflects the presence or absence of a coded diagnosis at the enrollment visit rather than biochemical eligibility. All enrolled patients met the inclusion criterion of having at least one documented sK^+^ value > 5.0 mmol/L within the 12 months prior to enrollment and were prescribed SZC on the basis of clinically confirmed hyperkalemia. The high proportion of patients categorized as “No diagnosis” in FAS-P1 therefore represents missing diagnostic coding at enrollment, not absence of hyperkalemia. As diagnostic coding and laboratory confirmation may occur at different timepoints in routine practice, this discrepancy does not indicate treatment of normokalemic patients but reflects variation in documentation across study sites.

### 3.3 Safety

The occurrence of AEs, SAEs, DAEs, and specific AEs is summarized in Table 2. Overall, 283 patients (64.0%) and 574 patients (65.4%) reported AEs with incidence rates of 2.9 (95% CI: 2.2, 3.8) and 181.2 (95% CI: 165.0, 199.0) per 100 person years in the FAS-P1 and FAS-P2, respectively. The most common AEs reported were related to edema and hypokalemia.

**TABLE 2:** Summary of AEs.

Adverse events	FAS-P1		FAS-P2	
	Total (N = 442), n (%)^1^	Incidence rate (95% CI), per 100 person-Years^2^	Total (N = 878), n (%)^1^	Incidence rate (95% CI), per 100 person- years^2^
Any AE	283 (64.0)	2.9 (2.2, 3.8)	574 (65.4)	181.2 (165.0, 198.9)
Adverse events as judged by the investigator to Be causally related to SZC	18 (4.1)	0.6 (0.3,1.1)	38 (4.3)	11.1 (7.6,16.2)
Any SAE	119 (26.9)	0.2 (0.1, 0.7)	270 (30.8)	77.4 (67.1, 89.3)
SAE as judged by the investigator to Be causally related to SZC	2 (0.5)	0	4 (0.5)	0.8 (0.2,3.3)
Any AE leading to drug permanently discontinued	6 (1.4)	0.1 (0.0, 0.4)	15 (1.7)	2.9 (1.4, 6.0)
Any specific AE				
Edema	13 (2.9)	0.1 (0.0, 0.4)	33 (3.8)	9.5 (6.3, 14.2)
Hypokalemia	23 (5.2)	0.2 (0.1, 0.7)	57 (6.5)	10.3 (7.0, 15.2)

^[1]^Patients with multiple events in the same category were counted only once in that category.

^[2]^The Incidence rate for the second period was calculated as the number of patients with events during the second period divided by the total person-time as treated by SZC, in the second period, where the end date was the SZC, dose stop date +1, the start date was the date of the second visit (start date of the second period) for new users, and the date of the first visit (study enrollment) for ongoing users. A 95% CI, was estimated following the Poisson distribution.

Abbreviations: AE, adverse events; CI, confidence interval; FAS-H, Full analysis set-hemodialysis; FAS-P1, Full analysis set-First Period; FAS-P2, Full analysis set-second period; SAE, serious adverse events.

Furthermore, SAEs were reported in 119 patients (26.9%) in the FAS-P1 with an end stage renal disease (n = 25, 5.7%), pneumonia (n = 13, 2.9%), and COVID-19 (n = 11, 2.5%) being the most common SAEs, whereas 270 patients (30.8%) reported SAE in the FAS-P2 with the highest incidence rate per 100 person-years being the end stage renal disease (13.2 [95% CI: 9.3, 18.6]), pneumonia (10.3 [95% CI: 7.0, 15.2]), and CKD (5.8 [95% CI: 3.4, 9.7]).

In the FAS-P1, 6 patients (1.4%) reported AEs leading to permanent drug discontinuation with COVID-19 (n = 3, 0.7%) being the most common DAE. In the FAS-P1 subgroups, no patient reported any AE leading to permanent drug discontinuation. In the FAS-P2, 15 patients (1.7%) reported AEs leading to permanent drug discontinuation and DAEs with the highest incidence rate per 100 person-years by PT were cardio-respiratory arrest (1.2 [95% CI: 0.4, 3.8]), COVID-19 (0.8 [95% CI: 0.2, 3.3]), and pneumonia (0.8 [95% CI: 0.2, 3.3]).

The specific AE incidence rate per 100 person-years in hypokalemia and edema peripheral was 0.2 (95% CI: 0.1, 0.7) and 0.1 (95% CI: 0.0, 0.4), respectively in the FAS-P1 and 10.3 (95% CI: 7.0, 15.2), and 4.5 (95% CI: 2.5, 8.2), respectively in the FAS-P2.

In the individual FAS-P1 subgroups, no patient reported any AE leading to permanent drug discontinuation. In the CKD stage 5/non-hemodialysis subgroup, 64 patients (66.0%) reported AEs, and 29 patients (29.9%) reported SAEs, which were the highest incidence rates among the different subgroups.

In the FAS-P1, 18 patients (4.1%) reported causally related AEs, and 2 patients (0.5%) reported causally related SAEs with no case of AEs leading to permanent drug discontinuation. However, in the FAS-P2, 38 patients (4.3%) reported causally related AEs and 4 patients (0.5%) reported causally related SAEs, with no patient deemed to have causally related AEs leading to the drug being permanently discontinued.

The incidence of AEs, as judged by the investigators to be causally related to the SZC treatment, was 0.6 (95% CI: 0.3, 1.1) per 100 person-years in the FAS-P1, 11.1 (95% CI: 7.6, 16.2) per 100 person-years in the FAS-P2, 7.7 (95% CI: 3.7, 16.1) in the FAS-P2-new user group, and 13.2 (95% CI: 8.5, 20.5) in the FAS-P2-ongoing user group.

### 3.4 Effectiveness

In the FAS-P1, the mean (SD) sK^+^ levels were 5.8 (0.6) and 5.0 (0.6) mmol/L at visit 1 and visit 2, respectively with a mean (SD) change of - 0.9 (0.8) mmol/L (95% CI: 0.9, - 0.8) in sK^+^ level. In the FAS-P2, the overall mean (SD) “within” patient sK^+^ levels were 5.1 (0.6) mmol/L (95% CI: 5.0, 5.1). In the ongoing user group and the new user group in the FAS-P2, the mean (SD) “within” patient sK^+^ levels were 5.0 (0.6) mmol/L (95% CI: 4.9,5.1), and 5.2 (0.6) mmol/L (95% CI: 5.1, 5.2) respectively. In the FAS-H, the mean (SD) “within” sK^+^ was 5.2 (0.6) mmol/L (95% CI: 5.1, 5.3).

The proportion of patients with normokalemia (sK^+^ level between 3.5 and 5.0 mmol/L) in the FAS-P1 at visit 2 was 51.3%, whereas the percentage of patients with sK^+^ levels 3.5–5.5 mmol/L at visit 2 was 79.5%. Similarly, the percentage of patients with normokalemia (sK^+^ level, 3.5–5.0 mmol/L) was 47.3% in the FAS-P2 and 37.2% in the FAS-H, whereas the percentage of patients with sK^+^ levels 3.5–5.5 mmol/L was 77.9% and 69.0% in the FAS-P2 and FAS-H, respectively. Effectiveness was also assessed in the overall cohort. Mean sK^+^ decreased from 5.8 mmol/L at baseline to 5.0 mmol/L at Visit 2, and remained stable throughout the maintenance period (mean 5.1 mmol/L), demonstrating consistent potassium control across the full population.

At visit 5, the proportions of patients with sK^+^ levels 3.5–5.0, 3.5–5.3, and 3.5–5.5 mmol/L were 49.5 (95% CI: 45.1, 53.9), 64.8 (95% CI: 60.5, 69.0), and 73.5 (95% CI: 69.4, 77.3), respectively, in FAS-P2. The proportions of normokalemic patients at 6 months with CKD stage at enrollment are shown in Table 3. A total of 2 out of 6 patients with stages 1–2 CKD and 39 out of 94 with stages 3–4 CKD had available sK^+^ measurements. In patients with stage 5 CKD, available sK^+^ measurements were observed in 66 out of 102 patients with hemodialysis and in 57 out of 115 patients without hemodialysis. Furthermore, patients were categorized into three different sK + levels, i.e., [3.5, 5.0], [3.5, 5.3], and [3.5, 5.5] mmol/L. The proportion of patients with sK^+^ level of [3.5, 5.0] mmol/L with stage 1–2 and 3–4 CKD was 50% (1.3, 98.7) and 59.0% (42.1, 74.4), respectively. In patients with stage 5 CKD with and without hemodialysis, the proportion was 51.5% (38.8, 64.0) and 63.2% (49.3, 75.5), respectively.

**TABLE 3:** Proportion of normokalemic patients at 6 months by CKD stage/hemodialysis at enrollment.

CKD stage/hemodialysis	1–2 (n = 6)	3–4 (n = 94)	5 without hemodialysis (n = 115)	5 with hemodialysis (n = 102)
Number of patients with available sK^+^ measurements	2	39	57	66
Patients with sK^+^ level of [3.5, 5.0] mmol/L				
Number of patients	1	23	36	34
Proportion, % (95% CI)	50.0 (1.3, 98.7)	59.0 (42.1, 74.4)	63.2 (49.3, 75.5)	51.5 (38.9, 64.0)
Patients with sK^+^ level of [3.5, 5.3] mmol/L				
Number of patients	1	25	43	44
Proportion, % (95% CI)	50.0 (1.3, 98.7)	64.1 (47.2, 78.8)	75.4 (62.2, 85.9)	66.7 (53.9, 77.8)
Patients with sK^+^ level of [3.5, 5.5] mmol/L				
Number of patients	1	28	45	52
Proportion, % (95% CI)	50.0 (1.3, 98.7)	71.8 (55.1, 85.0)	78.9 (66.1, 88.6)	78.8 (66.9, 87.9)

Abbreviations: CKD, chronic kidney disease; CI, confidence interval, sK^+^, serum potassium, n, no of patients.

The proportion of patients with an sK^+^ level of [3.5, 5.3 mmol/L] in stage 1–2 and 3-4 was 50.0% (1.3, 98.7) and 64.1% (47.2, 78.8), respectively. In stage 5 CKD with and without hemodialysis 66.7% (54.0, 77.8) and 75.4% (62.2, 85.9) patients, had sK^+^ levels of [3.5, 5.3] mmol/L, respectively. Furthermore, 50.0% of patients (95% CI:1.3, 98.7) with stage 1–2 CKD, and 71.8% of patients (95% CI: 55.1, 85.0) with stage 3–4 CKD had an sK^+^ level of [3.5, 5.5 mmol/L]. The proportion of patients with stage 5 CKD, with and without hemodialysis, exhibiting an sK^+^ level of [3.5, 5.5 mmol/L] was 78.8% (67.0, 87.9) and 78.9% (66.1, 88.6), respectively. It is important to note that the 6-month effectiveness outcomes reflect serum potassium values from patients who remained under follow-up and had available laboratory measurements at corresponding visits. Given the treatment discontinuation observed during the study, long-term potassium control results should be interpreted within the context of treatment persistence. Therefore, the 6-month findings represent real-world on-treatment effectiveness rather than a complete longitudinal outcome for all enrolled patients.

### 3.5 Treatment patterns

Patients in the FAS-P1, FAS-P2, and FAS-H received a mean (SD) dose of 9.5 (9.0), 4.2 (4.0), and 3.8 (5.9) g/day SZC, respectively. In the FAS-P1, 5 g QD (n = 186, 42.1%), 10 g TID (n = 83, 18.8%), and 5 g QOD (n = 48, 10.9%) were the most commonly used dosage and frequency as per the recommended dosage given on the local label whereas it was 5 g QD (n = 331, 37.7%), 5 g QOD (n = 112, 12.8%), and 10 g QD (n = 110, 12.5%) in the FAS-P2. The treatment dosage of SZC was similar between the new user group and the ongoing user group in the FAS-P2. In the FAS-H, 5 g QD (n = 40, 8.4%), 10 g TID (n = 25, 5.3%), and 10 g QD (n = 17, 3.6%) were the most commonly used dosage and frequency recommended dosage given on the local label (Table 4). Patients in the FAS-P1 and FAS-P2 with CKD stage 5 undergoing hemodialysis reported a mean daily dose of 14.6 (12.0) and 2.7 (2.0) g/day, respectively (Table 5).

**TABLE 4:** Summary extent of exposure.

Parameter category/statistic	FAS-P1 Total (N = 442)	FAS-P2 Total (N = 878)	Hemodialysis at enrollment subgroup analysis (FAS-H) total (N = 474)
Cumulative dose (g)			
n (missing)	438 (4)	854 (24)	457 (17)
Mean (SD)	30.4 (24.5)	285.8 (334.3)	217.9 (188.5)
Mean daily dose (g/day)^[2]^			
n (missing)	438 (4)	854 (24)	457 (17)
Mean (SD)	9.5 (9.0)	4.2 (3.9)	3.75 (5.9)
Dose frequency, n (%)^[3]^			
5 g QOD	48 (10.9)	112 (12.8)	3 (0.6)
5 g QD	186 (42.1)	331 (37.7)	40 (8.4)
10 g QD	43 (9.7)	110 (12.5)	17 (3.6)
10 g BID	20 (4.5)	51 (5.8)	8 (1.7)
10 g TID	83 (18.8)	71 (8.1)	25 (5.3)

Percentages were based on the number of patients from the FAS -P1, and FAS-P2 groups.

^[1]^Treatment duration (days) = Date of second visit- Date of first visit +1 - Days of interruption.

^[2]^Mean daily dose (g/day) = Cumulative dose (g)/Treatment duration (days).

^[3]^Categories were not mutually exclusive. Only the recommended dosage and frequency on the local label were shown here.

Abbreviations: BID, twice a day; FAS-H, Full analysis set-hemodialysis; FAS-P1, Full analysis set-first period; FAS-P2, Full Analysis Set-Second Period; QD, once a day; QOD, every other day; TID, three times a day.

**TABLE 5:** Summary of extent of exposure by CKD stage/hemodialysis at enrollment subgroup (FAS-P1 and FAS-P2).

Subgroups→	FAS-P1				FAS-P2			
Parameter Category/Statistic↓	CKD stage 1–2 (N = 3)	CKD stage 3–4 (N = 63)	CKD stage 5/non-hemodialysis (N = 97	CKD stage 5/Hemodialysis (N = 38	CKD stage 1–2 (N = 6	CKD stage 3–4 (N = 94	CKD stage 5/Non-hemodialysis (N = 115	CKD stage 5/Hemodialysis (N = 102
Treatment (days)^[1]^								
n (missing)	3 (0)	63 (0)	97 (0)	38 (0)	6 (0)	94 (0)	115 (0)	102 (0)
Mean (SD)	4.0 (0.0)	3.7 (0.8)	3.5 (0.8)	3.1 (1.1)	76.7 (80.9)	75.7 (69.5)	74.5 (68.5)	124.2 (71.9)
Median	4.0	4.0	4.0	3.0	53.0	41.5	44.0	169.0
Q1, Q3	4.0, 4.0	3.0, 4.0	3.0, 4.0	2.0, 4.0	7.0, 162.0	13.0, 151.0	13.0, 152.0	55.0, 184.0
Min, max	4, 4	1, 5	1, 4	1, 5	6, 179	1, 194	1, 214	1, 194
Cumulative dose (g)								
n (missing)	3 (0)	62 (1)	96 (1)	38 (0)	6 (0)	92 (2)	109 (6)	100 (2)
Mean (SD)	36.7 (15.3)	40.9 (22.9)	38.4 (23.0)	40.8 (34.9)	399.6 (486.5)	342.1 (447.1)	324.2 (388.8)	267.5 (183.0)
Median	40.0	37.5	32.5	40.0	218.8	192.5	191.4	276.8
Q1, Q3	20.0, 50.0	20.0, 55.0	20.0, 60.0	8.6, 60.0	40.0, 605.0	67.9, 458.8	35.0, 450.0	100.7, 396.4
Min, max	20.0, 50.0	10.0, 90.0	5.0, 120.0	4.3, 120.0	30.0, 1,285.0	5.0, 2790.0	5.0, 1820.0	2.9, 814.3
Mean daily dose (g/day)^[2]^								
n (missing)	3 (0)	62 (1)	96 (1)	38 (0)	6 (0)	92 (2)	109 (6)	100 (2)
Mean (SD)	9.2 (3.8)	11.6 (7.3)	12.7 (9.6)	14.6 (11.9)	6.6 (4.4)	5.5 (3.6)	5.8 (4.3)	2.7 (2.0)
Median	10.0	10.0	10.0	14.2	5.0	5.00	5.00	2.1
Q1, Q3	5.0, 12.5	5.0, 15.0	5.0, 16.2	2.1, 30.0	3.4, 7.9	2.9, 5.9	3.5, 7.1	2.1, 2.3
Min, max	5.0, 12.5	2.5, 30.0	1.3, 30.0	1.1, 30.0	3.4, 15.0	0.0, 20.0	0.0, 30.0	0.0, 15.0
Dose frequency, n (%)^[3]^								
5 g QOD	0	11 (17.5)	12 (12.4)	1 (2.6)	2 (33.3)	27 (28.7)	21 (18.3)	2 (2.0)
5 g QD	2 (66.7)	43 (68.3)	56 (57.7)	8 (21.1)	5 (83.3)	66 (70.2)	78 (67.8)	6 (5.9)
10 g QD	0	15 (23.8)	5 (5.2)	0	0	26 (27.7)	16 (13.9)	1 (1.0)
10 g BID	0	8 (12.7)	4 (4.1)	2 (5.3)	0	13 (13.8)	6 (5.2)	3 (2.9)
10 g TID	0	17 (27.0)	27 (27.8)	15 (39.5)	0	20 (21.3)	16 (13.9)	2 (2.0)

Percentages were based on the number of patients from FAS-P1, and FAS-P2.

^[1]^Treatment duration (days) = Date of second visit–Date of first visit +1 –day of interruption.

^[2]^Mean daily dose (g/day) = Cumulative dose (g)/Treatment duration (days).

^[3]^Categories were not mutually exclusive.

Abbreviations: BID, twice a day; CKD, chronic kidney disease; FAS-H, Full analysis set-hemodialysis; FAS-P1, Full analysis set-first period; FAS-P2, Full Analysis Set-Second Period; QD, once a day; QOD, every other day; TID, three times a day.

Drug interruption (55.7%, 49.5%, and 64.2%) was the most common dose change type in reported FAS-P1, FAS-P2, and FAS-H, respectively. The most common reasons for dose change were patient decisions (43.3%, 38.4%, and 49.8%, respectively) rather than AEs (4.3%, 5.2%, and 7.4% respectively; Table 6).

**TABLE 6:** Summary of dose change.

Category	FAS-P1		FAS-P2		FAS-H	
	No. of patients (%)^[1]^ (N = 442)	No. of dose changes (%)^[2]^	No. of patients (%)^[1]^ (N = 878)	No. of dose changes (%)^[2]^	No. of patients (%)^[1]^ (N = 474)	No. of dose changes (%)^[2]^
Any dose change	376	812	687	1744	317	542
Dose change type^[3]^						
Dose increased	82 (18.6)	109 (13.4)	217 (24.7)	302 (17.3)	61 (12.9)	67 (12.4)
Dose reduced	154 (34.8)	227 (28.0)	349 (39.7)	537 (30.8)	86 (18.1)	103 (19.0)
Drug interrupted	332 (75.1)	452 (55.7)	584 (66.5)	863 (49.5)	270 (57.0)	348 (64.2)
Drug permanently discontinued	24 (5.4)	24 (3.0)	41 (4.7)	42 (24)	24 (5.1)	24 (4.4)
Reasons for dose change						
Adverse event	NA	35 (4.3)	NA	91 (5.2)	NA	40 (7.4)
Surgery	NA	0	NA	0	NA	0
Subject forgot to take dose	NA	19 (2.3)	NA	37 (2.1)	NA	21 (3.9)
Subject decision	NA	352 (43.3)	NA	669 (38.4)	NA	270 (49.8)
Missing	NA	0	NA	0	NA	0

^[1]^Percentages were based on the number of patients in the FAS-P1, FAS-P2, and FAS-H.

^[2]^Percentages were based on the number of dose changes in the FAS-P1, FAS-P2, and FAS-H.

^[3]^Categories were not mutually exclusive.

^[4]^Other factors included a researcher’s decision, patient death, and the patient undergoing hemodialysis.

Abbreviation: FAS-H, Full analysis set-hemodialysis; FAS-P1, Full analysis set-first period; FAS-P2, Full analysis set-second period; NA, not applicable.

## 4 Discussion

This study provides a real-world evaluation of SZC, with safety as the primary assessed outcome and effectiveness and treatment patterns as secondary measures. The findings demonstrate that SZC was well tolerated and maintained potassium control across varied patient groups, including those receiving hemodialysis. The safety results observed in this study are consistent with international SZC clinical development data. In the HARMONIZE trial and its long-term extension studies, edema and hypokalemia were among the most commonly reported adverse events with a dose-dependent pattern, similar to the trends seen in the present cohort. Additionally, large real-world studies from the United States, Japan, and Europe have demonstrated comparable tolerability, supporting the global consistency of SZC’s safety profile across diverse CKD and heart-failure populations. From a clinical practice perspective, these findings reinforce the role of SZC as a practical potassium-lowering agent that can be used in both the correction-phase and the maintenance phase to support long-term potassium management. The stable sK^+^ levels observed over 6 months suggest that SZC may enable continued use of RAAS inhibitors, particularly beneficial in patients with CKD or heart failure, where hyperkalemia frequently limits optimal therapy. Such real-world consistency is important because treatment decisions in routine care are influenced by comorbidities, polypharmacy, and dialysis schedules, which differ from controlled clinical-trial conditions. The final results from the ACTUALIZE study were in line with the prior interim results and demonstrated the effectiveness of SZC in maintaining normokalemia in patients with HK by reducing sK^+^ levels with good tolerability profile (Shen et al., 2024). Overall, the treatment patterns of SZC in the real-world clinical practice for treating HK in Chinese patients generally followed the recommended dosage on the local label (average daily dose was 9.5 and 4.2 g/day in the FAS-P1 and FAS-P2 respectively).

In this study, SZC was well tolerated with no major concerns and the incidence of AEs was comparable across all groups. The AEs were mild to moderate in severity and manageable, similar to those reported in clinical trials. It is noteworthy to mention that AEs seldom contributed as reasons for dose change across all groups in this study. However, differing from the results of our interim analysis wherein GI AEs were commonly observed, the complete safety results of this study showed that edema (2.9% and 3.8%) and hypokalemia (3.8% and 6.5%) occurred most commonly in FAS-P1 and FAS-P2, respectively.

This observation was similar to other trials in both the correction and maintenance phases. The incidence of edema was dose-dependent (4.4%, 5.9%, and 16.1% of patients receiving SZC 5, 10, and 15 g/day, respectively) in a trial with a short treatment duration (≤28 days) (Lokelma, 2018) and was reported in 8% and 10% of patients during treatment for up to 11 months (Roger et al., 2019) and 12 months, respectively (Spinowitz et al., 2019). Generally, edema is more frequently associated with SZC 15 g/day (approved only for patients undergoing HD) and is attributed to the fact that with the absorption of sodium, there is a greater risk of fluid overload in patients with renal insufficiency or severe HF, both of which may occur with HK (Sodium zirconium cyclosilicate, 2018). Another commonly reported AE associated with SZC is hypokalemia as observed in the current study and previous studies, which could be attributed to being associated with higher SZC doses because of its mechanism of action (Hoy, 2018). Nevertheless, it is crucial to monitor HK when administering SZC at higher doses and take appropriate dose optimization steps to ensure patient safety in real-world conditions.

A reduction in sK^+^ levels was observed in patients with HK, between visit 1 and visit 2 with a mean change of −0.9 mmol/L, indicating effective homeostatic control in FAS-P1. In a phase 2 trial, SZC 10 g TID improved HK after 48–96 h in patients with sK^+^ levels of 5.0–6.0 mmol/L and stable stage 3 CKD (Ash et al., 2015). In line with previous studies, SZC showed improvement in HK condition in the correction-phase and consistently maintained normokalaemia in this study (Ash et al., 2015; Spinowitz et al., 2019).

Additionally, in FAS-P2 phase, ongoing and new users had a reduction in sK^+^ levels with a mean serum level between 5.0 and 5.2 mmol/L, respectively, indicating that sK^+^ levels continued to be maintained throughout the maintenance period. However, a higher proportion of ongoing users in the FAS-P2 maintained sK^+^ levels between 3.5 and 5.0 mmol/L, suggesting that the long-term use of SZC may help control sK^+^ levels. Corroborating our results, a phase 3 study underscored the efficacy of SZC in long-term maintenance of normokalemia up to 12 months, with 88% of participants achieving sK^+^ ≤ 5.1 mmol/L in 1 year (Spinowitz et al., 2019).

We also reported that 37.2% of patients were normokalemic in the FAS-H (sK^+^ levels: 3.5–5.0 mmol/L), whereas 69.0% of them had sK^+^ levels in the range of 3.5–5.5 mmol/L at visit 6. In a previous DIALIZE study, SZC was effective in the management of hyperkalemia in Chinese patients undergoing HD (37.3% of patients achieving normokalemia vs. 10.4% in the placebo group), which is consistent with previous research, which showed that SZC treatment reduced potassium levels in patients with CKD, regardless of the stage of their disease (Spinowitz et al., 2019).

Potassium control targets in this study reflect thresholds commonly applied in Chinese clinical practice (3.5–5.3 or 3.5–5.5 mmol/L), which tend to be slightly higher than the strict 3.5–5.0 mmol/L range used in many international trials. When interpreted alongside data from global studies, the findings indicate that SZC effectively maintains potassium within clinically accepted ranges across different practice environments. This alignment suggests that therapeutic performance is consistent despite regional variation in monitoring intervals and potassium thresholds. In doing so, the study aimed to reflect actual clinical practices and accommodate varying standards for potassium level reduction across study sites. The results of the study therefore suggested that SZC can effectively control long-term sK^+^ levels at around 5.0 mmol/L in the real-world practice in China.

The primary limitation of this study is its observational nature with confounding factors and biases, which could influence the study interpretations such as uncontrolled patient characteristics and recall bias. Furthermore, the study assesses the real-world scenarios that may result in less stringent follow-ups, treatment adherence, and compliance compared with controlled interventions. In addition, the study lacked the controls because this was a single arm thereby impacting and limiting the interpretation of the study results. Additionally, the observed discontinuation rate may influence the interpretation of 6-month effectiveness outcomes, as these data primarily represent patients who continued SZC therapy and remained under follow-up, which may introduce attrition-related bias. As an observational study, several potential confounders may influence the outcomes. These include comorbidities such as diabetes, CKD stage, and heart failure; concomitant RAAS-inhibiting medications; dialysis status; baseline potassium variability; and differences in treatment adherence. While the study design captures these real-world complexities, they may affect both safety and effectiveness interpretations. To mitigate this, subgroup analyses (including the hemodialysis cohort) were provided, and laboratory values were summarized separately for each analysis set.

## 5 Conclusion

In this real-world study, the safety profile of SZC was consistent with observations from controlled clinical trials, with no new safety signals identified. SZC effectively reduced serum potassium during the early correction-phase and maintained potassium levels within clinically acceptable ranges throughout the maintenance phase. The treatment patterns observed in routine practice aligned with the approved dosing recommendations. As the study did not evaluate SZC for emergency management, the findings should be interpreted within the context of non-acute, real-world use. Overall, SZC represents a suitable option for ongoing potassium management in patients with hyperkalemia in clinical practice.

## Data Availability

The original contributions presented in the study are included in the article/Supplementary Material, further inquiries can be directed to the corresponding author.
