# Supplementary material for: Safety, effectiveness and treatment patterns of sodium zirconium cyclosilicate for hyperkalemia management in China: actualize study
Source: Front Pharmacol. 2026 May 14;17:1744687. doi: 10.3389/fphar.2026.1744687 (PMC13216492; doi:10.3389/fphar.2026.1744687)
Supplement: Supplementary file 1 [file Table1.docx]

**Table S1. Additional Comorbidities and Concomitant Therapies at Enrollment**

| **Comorbidities** | | | |
| --- | --- | --- | --- |
| **Category** | **FAS-P1 Total (N=442),**  **n (%)** | **FAS-P2 Total (N=878),**  **n (%)** | **FAS-H Total (N=474),**  **n (%)** |
| Chronic kidney disease (CKD) | 215 (48.6) | 385 (43.8) | 146 (30.8) |
| Diabetes mellitus | 41 (9.3) | 94 (10.7) | 41 (8.6) |
| Heart failure | 71(16.1) | 171 (19.5) | 103 (21.7) |
| Endocrine disorders | 177 (40.0) | 425 (48.4) | 306 (64.6) |
| Nervous system disorders | 123 (27.8) | 257 (29.3) | 137 (28.9) |
| Gastrointestinal disorders | 74(16.7) | 182 (20.7) | 107 (22.6) |
| Respiratory, thoracic and mediastinal disorders | 60(13.6) | 131 (14.9) | 59 (12.4) |
| Congenital, familial and genetic disorders | 25 (5.7) | 40 (4.6) | 26 (5.5) |
| **Concomitant Therapies** | | | |
| Calcium Channel Blockers | 339 (76.7) | 670 (76.3) | 359 (75.7) |
| Beta-blockers (β-blockers) | 218 (49.3) | 412 (46.9) | 224 (47.3) |
| Drugs Used in Diabetes | 195 (44.1) | 349 (39.7) | 149 (31.4) |
| Agents Acting on The Renin-Angiotensin System | 192 (43.4) | 387 (44.1) | 233 (49.2) |
| Antithrombotic Agents | 187 (42.3) | 365 (41.6) | 230 (48.5) |
| Loop diuretics | 162 (36.7) | 267 (30.4) | 64 (13.5) |
| Vitamins | 150 (33.9) | 347 (39.5) | 221 (46.6) |
| Mineral Supplements | 138 (31.2) | 259 (29.5) | 145 (30.6) |
| Lipid-modifying agents | 136(30.8) | 281 (32.0) | 99 (20.9) |
